# Combined effusive-explosive silicic volcanism straddles the multiphase viscous-to-brittle transition

**DOI:** 10.1038/s41467-018-07187-w

**Published:** 2018-11-08

**Authors:** Fabian B. Wadsworth, Taylor Witcher, Caron E. J. Vossen, Kai-Uwe Hess, Holly E. Unwin, Bettina Scheu, Jonathan M. Castro, Donald B. Dingwell

**Affiliations:** 1 Department of Earth Sciences, Durham University, Durham, DH1 3LE UK; 2 Department of Earth- and Environmental Science, Ludwig-Maximilians-Universitat, Theresienstr. 41, 80333 Munich, Germany; 3 Department of Earth Sciences, University of Oxford, South Parks Road, Oxford, OX1 3AN UK; 4 Institute of Earth Sciences, University of Mainz, Becherweg 21, 55116 Mainz, Germany

## Abstract

Magma is a viscoelastic fluid that can support fracture propagation when local shear stresses are high, or relax and flow when shear stresses are low. Here we present experiments to confirm this using synthetic and natural magmatic liquids across eruptive conditions and use Maxwell’s linear viscoelasticity to parameterize our results and predict the maximum stresses that can be supported during flow. This model proves universal across a large range of liquid compositions, temperatures, crystallinity and rates of strain relevant to shallow crustal magma ascent. Our results predict that the 2008 Volcán Chaitén eruption resided in the viscous field at the onset of magma ascent, but transitioned to a mixed viscous-brittle regime during degassing, coincident with the observed combined effusive-explosive behaviour during dome extrusion. Taking a realistic maximum effusive ascent rate, we propose that silicic eruptions on Earth may straddle the viscous-to-brittle transition by the time they reach the surface.

## Abstract (web summary)

Recent observations of silicic eruptions show that they can be both effusive and explosive at the same time. Here the authors use scaled experiments to demonstrate that magma in effusive eruptions will fracture during flow to the Earth’s surface, accommodating mixed eruption styles.

## Introduction

An apparent dichotomy exists in volcanic eruptions on Earth between events that are dominantly effusive—producing lavas—and those that are explosive in character—producing volcanic ash fragments in plumes or fast-moving density currents^1–6^. However, these are end member descriptions and single eruptions can transition from one style to another^4–7^, or exhibit combined effusive-explosive behaviour simultaneously^8,9^. These differences in eruptive behaviour cannot be attributed to variations in primary volatile contents at storage conditions, nor to magma composition alone, and instead are dominantly controlled by the details of ascent dynamics^3,10–13^. Indeed, magmas can undergo local brittle fracturing in otherwise effusive events^11^.

Some of the most spectacular natural field examples from which we can infer this combined behaviour—fracture-formation during otherwise viscous flow of magma—include the preserved Mule Creek volcanic vent^14^ (New Mexico, U.S.), and Torfajökull^15,16^ or Hrafntinnuhryggur^17^ (Iceland). Recently, direct observations of combined explosive-effusive styles have been made at eruptions of Santiaguito volcano (Guatemala) from 2007 to 2015^18^, and at two eruptions in Chile: Volcán Chaitén in 2008^7,19^ and Cordón Caulle from 2011 to 2013^8,20^. In these events, detailed documentation could be made of fractures opening through an effusive lava flow or dome lava, and acting as gas-escape pathways and explosive ash vents^8,18^. When such eruptions end, these fractures are often locally welded shut, or filled with ash and pyroclasts that have sintered back together, demonstrating that these fractures formed and then closed all while ambient temperatures remained high^16^.

Local or wholesale fracturing of hot magma occurs because elastic stresses accumulate in the volcanic liquid when the rates of deformation are high^1,11,21–23^. This view of magma rheology is supported by the occurrence of volcanic rocks that can be shown to have fractured and rehealed while hot^14,16,24,25^, experimental evidence demonstrating the feasibility of breaking magmatic liquids or suspensions at eruptive temperatures^21,23,26^, and numerical simulations of fracturing in viscoelastic magma during flow^11,27^. Maxwell proposed the simplest model of how elastic shear stresses are stored or relaxed in fluids; such that relaxation occurs over a characteristic time *λ*_*r*_ = *μ*/*G*_∞_ where *μ* is the liquid viscosity (Pa.s) and *G*_∞_ is the elastic shear modulus (Pa)^1,28,29^. We can compare this time to the time characteristic of flow $\lambda = 1/\dot \gamma$ where $\dot \gamma$ is the shear strain rate (s^−1^). This balance between forced flow and relaxation of stress yields a dimensionless strain rate, or a scale-independent Weissenberg number

1 $${\mathrm{Wi}} = \frac{{\lambda _r}}{\lambda } = \frac{\mu }{{G_\infty }}\dot \gamma$$

such that Wi ≫ 1 is a first-order constraint of the unrelaxed brittle field, while Wi ≪ 1 represents the viscous field. The value of *G*_∞_ is approximately 10^10 ^Pa and broadly independent of composition or temperature^28,30^. Viscosity *μ*, however, is strongly dependent on temperature *T* and can be predicted using empirical models that additionally account for liquid composition including its dissolved volatile concentrations $C_{{\mathrm{H}}_2{\mathrm{O}}}$ (in wt.%; where total dissolved water is the dominant volatile phase in most shallow magmas)^31^. Therefore, knowing *G*_∞_ and $\mu (T,C_{{\mathrm{H}}_2{\mathrm{O}}})$, we can predict *λ*_*r*_ for any magmatic liquid at any eruptive temperature.

We explore the efficacy of Eq. 1 in scaling transitions from viscous to elastic behaviour by conducting experiments in a large-scale press in which we load a cylinder of synthetic or natural volcanic glass, heat it to a set temperature, and compress it in the direction parallel to the axis of rotational symmetry. We control the upper piston velocity at a constant value, which confers a constant axial strain rate $\dot \gamma _a$, and we record the evolution of axial stress *σ* (computed from axial force). We refer the reader to the methods section for full details of the experimental technique, equipment, the calibration at low Wi, uncertainties, and data treatment details. We characterize the response of the liquid as viscous if the stress stabilizes to a steady state after an initial transient, as brittle if the stress linearly increases with time toward a single large drop to zero stress, or as transitional between these two end-member behaviours if the stabilisation to a steady state is interrupted by drops in stress.

## Results

### Healed brittle fractures in effusive or combined explosive-effusive volcanic events

**Fig. 1:**
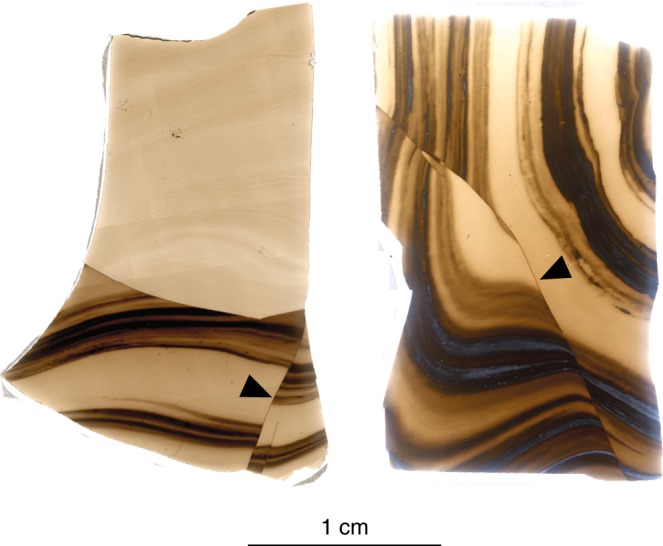
Photomicrographs of obsidian from Hrafntinnuhryggur, Krafla volcano (Iceland) collected in situ toward the margins of an effusive dyke-geometry conduit^17^ with textural evidence for fracture formation and subsequent healing while at high temperature (marked with arrows). These images were taken by Jenny Schauroth

There is a large body of textural evidence for brittle fractures in silicic magma that have healed, all while temperatures remain high^14,16,24,25,32,33^. In Fig. 1, we provide an example from Hrafntinnuhryggur, in Krafla, Iceland (photo taken by Jenny Schauroth). Other examples have been documented in the products of both dominantly effusive^11,17^, and mixed explosive-effusive^24^ silicic eruptions. These healed, or partially healed, fractures often contain welded fragments^9,34^. We take these observations as a motivating starting point in the design of our experimental campaign to understand the conditions required for fracture formation in otherwise viscous magma.

### Experimental results

We map our experimental data according to the response of the liquid in the regime space between *λ* and *λ*_*r*_, such that curves of constant Wi can be plotted. We additionally reanalyze published results for single-phase liquids for which sufficient detail was available to compute *λ*_*r*_ and *λ* (refs. ^22,26,35^), and map them in this same regime space (we use a constant *G*_∞_ = 10^10 ^Pa after refs. ^28,30^). Since the Weissenberg number accounts for the temperature- and compositional dependence via the incorporation of *μ*, and in the absence of any other time-dependent process, the transition from viscous to brittle behaviour will occur at a single Wi. Our experiments imply an important threshold at a critical Wi_c_ ~ 10^−2^, and thus delineate macroscopically viscous (Wi < 10^−2^) from macroscopically brittle results (Wi > 10^−2^). If Wi is increased further, then brittle behaviour is localized in a single explosive fracture event in the system, leading to a single large stress drop for Wi ≥ 0.04, which represents the dimensionless strain rates required for strain localization in a confined sample, or thorough shattering of the glass in an unconfined sample.

### Viscous dissipation as heat

**Fig. 2:**
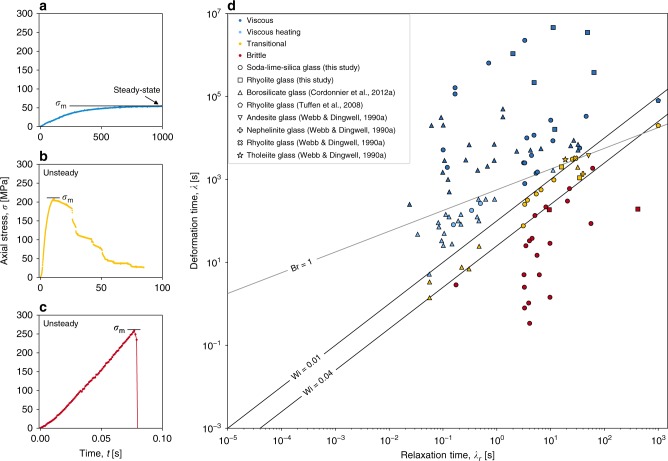
A regime diagram for the brittle failure of viscoelastic magmatic liquids. **a**–**c** Constitute the map key (or legend) for navigating through frame **d** and indicate typical behaviours cast as the evolution of an axial loading stress *σ* at high, moderate and low timescales of deformation *λ*, producing a **a** viscous, **b** transitional viscous-brittle, and **c** purely brittle response to loading, respectively. **d** A regime diagram in which experiments at constant *λ* are plotted as a function of the relaxation time *λ*_*r*_ and coloured according to the macroscopic response observed (see map key at left **a**–**c**). Results of this study are compared with a reanalysis of previous experimental results for magmatically-relevant pure liquids^22,26,35^. All data are consistent with viscous behaviour at Wi < 0.01 and brittle behaviour at ${\mathrm{Wi}} \,\,\gtrsim \,\,0.04$. Where *G*_∞_ was not known, we have taken 10^10 ^Pa for silicate liquids^28,30^

There exists an additional process in which heat is produced by internal friction in flowing viscous liquids, which is parameterized here by the power density ${\mathrm{\Phi }}_{\mathrm{g}} = 2\mu \dot \gamma ^2$. Such heat can be conductively lost out of a system of a given size with a power density $\Phi_l = \nabla q = \kappa \nabla^2 T$ where *q* is the heat flux (Wm^−2^ ) out of the system of a given area, and *κ* is the thermal conductivity (W m^−1^ K^−1^). The ratio of these powers is a Brinkman number Br = Φ_g_/Φ_l_ such that Br>1 yields net heat gains due to viscous dissipation as heat^22,36^. We can constrain this for our system, showing that for high *λ*_*r*_ (high *μ*), the samples will fracture before the accumulation of heat will occur. By contrast, at low *λ*_*r*_, heat storage prior to brittle behaviour will occur, potentially reducing *λ*_*r*_ further and pushing the system away from the critical Wi = 10^−2^. As Br is scale dependent, these regimes (of heat retention vs. dissipation) shift with system size. Here for our experimental data, we can plot the threshold Br = 1, calibrating Φ_l_ ≈ 10^4.5^W m^−3^ for the scale of our samples and set-up^22^ (Fig. 2). Data that showed a smooth, monotonic, time-dependent decrease in the peak stresses reached, can be characterized as heating by viscous dissipation, and are consistent with the region of Fig. 2d in which Br > 1 (below the line Br = 1 in this space).

### Extensions to account for multiphase magma

**Fig. 3:**
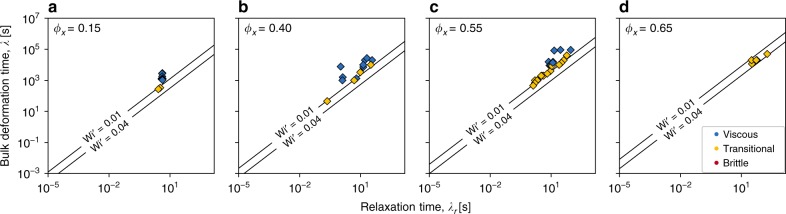
Scaling approaches to account for multiphase crystal-bearing magma. Here the timescales plotted on each axis are unscaled, while the critical Wi curves are scaled for the effect of crystals in order to demonstrate how the viscous-to-brittle transition shifts with increasing crystal content (**a**–**d**). When the axes are scaled instead, the data should be comparable regardless of crystal effects (see Fig. 4)

Magmas are rarely single-phase liquids. Two-phase crystal-bearing or bubbly magmas generally break at lower applied stresses or strain rates than the pure liquid phase^21^. To test this effect, we compile data from published sources^21^ collected using an analogue for rhyolitic magmas that contained crystals, and sheared in a simple-shear, torsional Paterson press. We find that in the case of crystal-bearing magmas, we can account for this effect by scaling the critical Weissenberg number Wi_c_ for the effect of crystals, by defining the two-phase critical Weissenberg number as ${\mathrm{Wi}}_{\mathrm{c}}^\prime = {\mathrm{Wi}}_{\mathrm{c}}(1 - \phi _x/\phi _m)$ where *ϕ*_*x*_ and *ϕ*_*m*_ are the crystal volume fraction, and the maximum packing crystal volume fraction, respectively. Following Cordonnier et al.^21^ we find that we must exchange *μ* for *η* in the definition of Wi_c_, where *η* is the measured suspension viscosity. The efficacy of this result is shown in Fig. 3 where *ϕ*_*m*_ = 0.74 for the moderately polydisperse crystal size distribution is given in ref. ^21^. Figure 3 demonstrates that ${\mathrm{Wi}}_{\mathrm{c}}^\prime$ is shifted to higher *λ* as the fraction of crystals is increased (e.g. compare Fig. 3a with Fig. 3d).

**Fig. 4:**
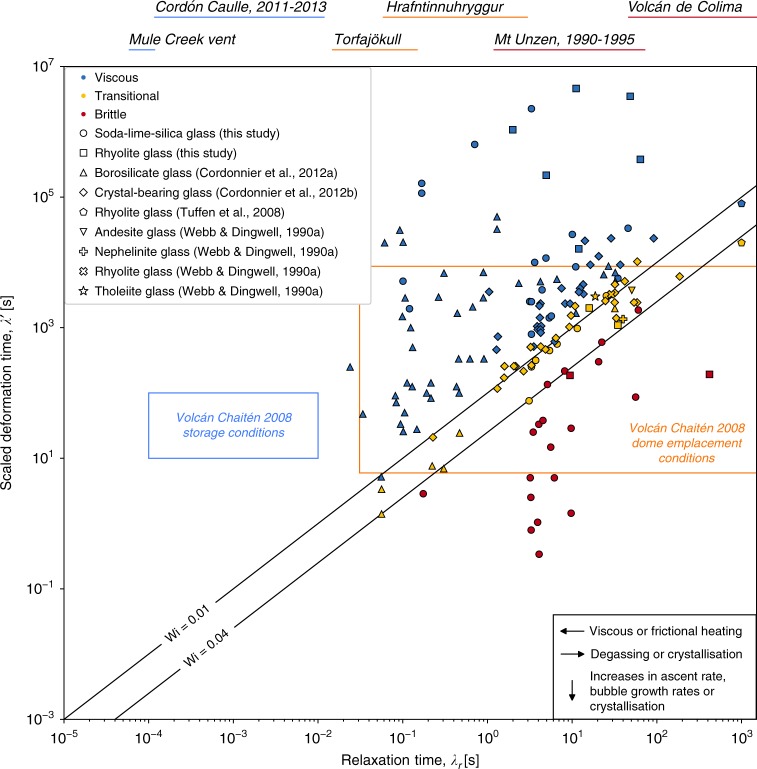
The universal map for understanding the conditions under which multiphase magma will fracture. The data from Figs. 2 and 3 are compiled and repeated here where the multiphase data from Fig. 3 is rescaled to be unified with all single-phase data (see text). The boxes represent two constraints of the 2008 eruption of Volcán Chaitén where *λ* is calculated from estimates of magma ascent rate *u* assuming *λ* = *L*/*u* where *L* is the conduit radius^7^. The blue box is calculated using the storage and shallow ascent conditions of $1 \le C_{{\mathrm{H}}_2{\mathrm{O}}} \le 4$ wt.% ascending at 0.5 m s^−1^ at *T* ~ 825 °C with no observable evidence for brittle behaviour prior to fragmentation^7^ and the yellow box is calculated using the range of surficial dome lava conditions of $0.15 \le C_{{\mathrm{H}}_2{\mathrm{O}}} \le 0.4$ wt.%^24^ extruding at up to 0.1 m s^−1^ vertically^41^ and exhibiting mixed explosive-effusive behaviour. Conduit radius is 5 m in both cases. For comparison, we give the range of *λ*_*r*_ for silica-rich aphyric and crystal-bearing magmas involved in a range of mixed explosive-effusive eruption styles. These ranges are grouped depending on whether *λ*_*r*_ is estimated using hydrous storage conditions (black; Cordón Caulle, Mule Creek vent), degassed surface conditions (dark grey; Torfajökull, Hrafntinnuhryggur), or degassed crystal-bearing dome lavas (pale grey; Mt Unzen, Volcán de Colima). The 2011–2013 eruption of Cordón Caulle involved magma with $2.5 \le C_{{\mathrm{H}}_2{\mathrm{O}}} \le 4.5$ wt.% at storage conditions of *T* ~ 896 °C^20^. The Mule Creek vent obsidian has estimated $2.5 \le C_{{\mathrm{H}}_2{\mathrm{O}}} \le 3$ wt.% emplaced at *T* ~ 800 °C^14^. The Torfajökull obsidian (Bláhnúkur) preserves low $0.27 \le C_{{\mathrm{H}}_2{\mathrm{O}}} \le 0.58$ wt.%, and was emplaced at *T* ~ 800 °C^15^. The 1990–1995 dome-forming magma from Mt Unzen erupted nominally dry at 780 ≤ *T* ≤ 880 °C with crystallinities 0.25 < *ϕ*_*x* _< 0.5^39^. The dome magma at Volcán de Colima erupted nominally dry at *T* ~ 940 °C with 0.4 < *ϕ*_*x* _< 0.6^40^. In all cases, rhyolitic liquid viscosities $\mu (T,C_{{\mathrm{H}}_2{\mathrm{O}}})$ are computed^31^ with the additional scaling with (1 − *ϕ*_*x*_/*ϕ*_*m*_)^−2^ where appropriate using Mueller et al.^38^ and a smooth crystal aspect ratio of 2, and converted to *λ*_*r*_ or $\lambda _r^\prime$ by taking *μ*/*G*_∞_ or *η*/*G*_∞_, respectively

An equivalent way to scale for the presence of crystals is by making substitutions in the definitions of *λ*_*r*_ and *λ*, redefining them as $\lambda _r^\prime = \eta /G_\infty$ and $\lambda \prime= 1/\dot \gamma \prime = (1 - \phi _x/\phi _m)\dot \gamma ^{ - 1}$. Here γ′ is the strain rate in the liquid amplified locally as liquid has to move more rapidly around crystals for a given bulk strain rate on the system $\dot \gamma$ as described above. This results in a critical Weissenberg number as ${\mathrm{Wi}} = \lambda _r^\prime /\lambda \prime$, which is equivalent to the Wi for single-phase liquids, and permits all data to be plotted together in a master regime diagram for multiphase and single-phase magmas where the breaking point Wi_c_ is at a constant position (Fig. 4).

The same scaling approach as above has been attempted for analogues of bubbly magma that are rapidly decompressed^37^. Here, the characteristic decompression time appears to be a first-order constraint of *λ*, separating results in which the bubbly samples expanded viscously from those which fragmented by bubble-overpressure at Wi ~ 10^−2^, as predicted by our experimental constraints. However, in detail, *λ* has a more complex relationship with decompression rate, and would require further work to scale across all Wi.

### A universal map for shear fractures in magma

In light of the multiphase constraint presented above, we can collapse the data for crystal-bearing, and single-phase liquid samples to the same regime map given in Fig. 2, when *λ*_*r*_ is replaced by $\lambda _r^\prime$ and *λ *by *λ*′ in the case of multiphase magmas. This gives a unified description in which all magma begins to break as Wi increases above 10^−2^ (Fig. 4).

### A framework for predicting shear-fracture in silicic eruptions

In this framework, some observations at silicic volcanoes can be tied to specific physical quantities via Eq. 1 with predictive power. To give a range of examples, we compile the range of *λ*_*r*_ we can calculate for individual eruptions in which some transitional explosive-effusive behaviour has been observed. We do this for three ‘families’ of silicic eruptions. (1) Eruptions for which the bulk of the constraints of $\mu (T,C_{{\mathrm{H}}_2{\mathrm{O}}})$ are for estimations of the magma at storage conditions in the crust and during the onset of ascent (Fig. 4, blue labels; eruptions plotted are 2011–2013 Cordón Caulle^20^ and Mule Creek vent^14^). (2) Eruptions for which the constraints of $\mu (T,C_{{\mathrm{H}}_2{\mathrm{O}}})$ are for the degassed magma at or near the Earth’s surface (Fig. 4, orange labels; eruptions plotted are Torfajökull^15^ and Hrafntinnuhryggur^17^). And (3) eruptions of degassed semi-crystalline dome-forming magmas for which *μ* is replaced with *η* and is additionally a function of *ϕ*_*x*_/*ϕ*_*m*_ following the model by Mueller et al.^38^ (Fig. 4, red labels; eruptions plotted are Mt Unzen^39^ and Volcán de Colima^40^; we use *ϕ*_*m*_ for smooth crystals with an average aspect ratio of 2 [ref. ^38^]). See the caption to Fig. 4 for more details.

Using our analysis, the critical deformation time *λ*_*c*_ or critical scaled deformation time $\lambda _c^\prime$ can be predicted for all eruptions of these types, thresholding the minimum deformation rates associated with the onset of brittle behaviour, or associated with mixed behaviour that is locally brittle. Moreover, as all magmas degas during ascent and eruption, producing spatially heterogeneous, and generally increasing *μ* (and therefore an increasing *λ*_*r*_), Fig. 4 demonstrates how the resultant range of relaxation times and evolution thereof would confer drastically different deformation behaviour and almost inevitably results in brittle behaviour during ascent and degassing.

A detailed look at the 2008 eruption of Volcán Chaitén can provide further insights. Using the temperature (825 °C during initial ascent^7^), water contents^24^ and average strain rates constrained for the magma storage conditions and initial propagation to the surface^7^, we can provide a range of possible *λ*_*r*_ and *λ* for the early phase of the eruption. This range plots in the viscous field (Fig. 4). This indicates that conduit-scale shear fracturing was not likely to be a dominant component of the initially Plinian explosive phase of the eruption. In this initial phase, the classical magma fragmentation by bubble overpressure process is likely dominant. As the eruption progressed, the magma degassed, and combined explosive-effusive behaviour resulted in dome extrusion punctuated by ash-venting^9,19,41^. Similar behaviour has been inferred to have occurred during silicic eruptions elsewhere^42^. Using the water contents measured in the dome lava^24^ and the dome extrusion rates^41^, we can compute a new range of *λ*_*r*_ and *λ* for the mid-stages to late-stages of the eruption, which straddles the critical Weissenberg number threshold (Fig. 4). This analysis implies that even as contiguous magma reached the surface, and the bulk of the explosive behaviour ceased, conduit-scale brittle fractures could open up, which is consistent with the observation of combined explosive-effusive eruption styles.

To generalize beyond a single eruption, we use the observation that constraints of silicic (rhyolitic and dacitic) magma ascent rates at most eruptions delineate dominantly explosive eruptions from effusive eruptions^43^. We note that the ascent rate that separates one eruptive style from another occurs around $0.01 \,\,< \,\,u \,\,< \,\,0.5\,{\mathrm{m}}.{\mathrm{s}}^{ - 1}$ (ref. ^43^), which therefore represent the maxima for an effusive eruption. As an approximate scaling, we note that the average conduit strain rate $\langle \dot \gamma \rangle$ will be $\langle \dot \gamma \rangle \approx u/L$, where *L* is the conduit radius. For bracketing values $5 \,\,< \,\,L \,\,< \,\,50$ m, we can predict a wide range of possible $\langle \dot \gamma \rangle$ for silicic eruptions, which in turn, give a range of $10 \,\,< \,\,\langle \lambda \rangle \,\,< \,\,5000$. Using Fig. 4 as a guide, we see that for silicic magmas ascending with this range of deformation timescales, will dominantly be in the viscous field ${\mathrm{Wi}} \ < \ 10^{ - 2}$ when close to their shallow storage conditions. However, using the range of *λ*_*r*_ plotted for silicic eruptions at degassed or partially crystallized conditions equivalent to the shallowest portions of final ascent, the same deformation timescales clearly make brittle behaviour very likely, even for the upper end of possible conduit radii and using the conservative estimates of $\langle \dot \gamma \rangle$. We therefore hypothesize that ascent-driven locally brittle behaviour at silicic volcanoes is common, and may be a key process in the recently observed combined explosive-effusive behaviour.

**Fig. 5:**
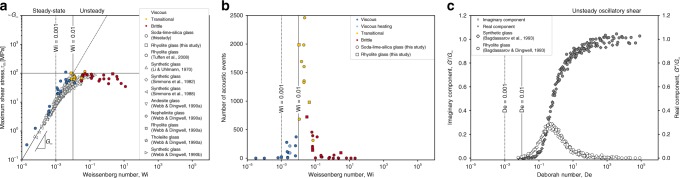
The consequence of viscoelasticity for the emplacement behaviour of magmas. **a** At Wi < 10^−2^, the maximum shear stress is *τ*_*m*_ = *G*_∞_Wi, and at Wi > 10^−2^, *τ*_*m*_ = 10^−2^*G*_∞_, which in the case of magmatic liquids is $\tau _m = {\cal O}(10^2)$ MPa, consistent with a reanalysis of data for synthetic^28, 44–46^ and natural silicate liquids^35^. Here *τ*_*m*_ is computed from *σ*_*m*_ using the Trouton ratio for cylindrical geometry where appropriate. **b** The acoustic event number confirming fracture-formation during deformation of magmatic liquids at ${\mathrm{Wi}} \,\,\gtrsim\,\, 3 \times 10^{ - 3}$ and dominant acoustic activity at Wi = 0.01. **c** The viscoelasticity of a rhyolitic liquid oscillated at forcing frequency *ω* to low strain amplitudes, yielding a real *G*′ and imaginary *G*″ component of the elastic modulus^47, 48^ as a function of the Deborah number De = *ωλ*_*r*_ and showing the onset of elasticity at De = 10^−2^, consistent with all data (Figs. 2 and 4)

In our experiments, the bulk axial stress *σ* is approximately related to the mean shear stress *τ* by *τ* = *σ*/3 (by using the Trouton ratio^28^). If we constrain our analysis to the regime space Br < 1, we can demonstrate that in the viscous regime, the peak shear stress value *τ*_*m*_ achieved in any experiment at steady state, scales with Wi via *τ*_*m*_ = *G*_∞_Wi predicted for viscoelastic liquids (via Eq. 1 assuming $\tau _m = \mu \dot \gamma$ in the low-${\mathrm{Wi}}$ regime). In the brittle regime, $\tau _m$ is broadly independent of Wi and is given by $\tau _m = 10^{ - 2}G_\infty$, which for magmatic liquids is $\tau _m = {\cal O}(10^8)$ Pa, consistent with the order of magnitude for the strength of glassy materials^28,44^. Using Eq. 1, we can additionally reanalyze published experimental data collected using a large range of silicate glass-forming synthetic and natural magmatic liquids^26,28,35,44–46^, all collected over a wide range of temperatures, demonstrating consistency with our analysis (Fig. 5a).

Our hypotheses are supported by observing the signatures of acoustic emission events—representing episodes of fracture nucleation and rapid growth. These increase above background noise at ${\mathrm{W}}{\mathrm{i}} \,\,\gtrsim \,\,3 \times 10^{ - 3}$, and become dominant at the threshold beyond which we observe macroscopic brittleness at Wi > 10^−2^ (Fig. 5b). Time-dependent drops in stress indicating brittleness are not directly observed in our macroscopic mechanical data as low as Wi = 10^−3^ (Fig. 2), possibly because the damage rate is low and small nucleating fractures are partially healing on the timescale of deformation. While our AE observations potentially lower the critical Wi_c_, we note that our use of cylindrical samples, for which the Trouton ratio for shear stresses and strain rates is only an approximate correction, make this difficult to interpret at this resolution.

We have found that, in general 0.01 < Wi < 0.04 represents a transitional window from viscous to brittle behaviour. To understand the Wi thresholds quantitatively, we compile data for viscous volcanic liquids deformed in the frequency domain where the amplitude of strain was sufficiently low to avoid fracturing^47,48^. In these experiments an oscillation of strain at forcing frequency *ω* results in a measured evolution of shear stress. In turn, this can be decomposed into the real *G*′ and imaginary *G*″ components of the elastic modulus. The normalized frequency *ωλ*_*r*_ is a Deborah number (the frequency domain counterpart to the Weissenberg number^49^), collapsing all data to a temperature-independent description. Such data demonstrates that the onset of effective non-zero accumulation of elastic stress also occurs at De ~ 10^−2^ (Fig. 5c), consistent with changes in *τ*_*m*_(Wi), acoustic emission data, and the brittle onset (Figs. 2 and 4).

Herein, we provide a non-dimensional framework for understanding the onset of brittle shear fractures in silicic magmas, accounting for multiphase heterogeneities of suspended crystals. Through the application of this framework to a few type-localities worldwide, including two recently observed rhyolitic dome-forming eruptions, we show that it is likely that shear fracturing is an important process in the shallow portions of effusive magma ascent with concomitant degassing. At storage conditions and the early parts of ascent, most silicic magmas appear to reside at conditions amenable to viscous behaviour without brittleness, however, we show that it is almost an inevitability that during ascent and degassing of volatiles, magmas will straddle the viscous-to-brittle transition at the Earth’s surface. Therefore, models of magma ascent that include the acknowledgment that ascent can be accommodated by sliding on fracture networks, are preferred over those that omit this. Finally, an implication of this framework is that most surficial silicic magmas will have undergone the transition to fracture-hosted open-system dynamics in which parts of the gas phase is strongly decoupled from the residual magma.

## Methods

### A high-temperature, high-load uniaxial press for magma deformation

We use a uniaxial compression press built by Voggenreiter GmbH. A linear variable differential transformer (LVDT) with a resolution of 10^−6^ m and a total 150 mm travel range is used with a hydraulic motor system to control the motion of the top piston. Within the working range 8.3 × 10^−7^ ms^−1^ to 1.0 × 10^−2^ ms^−1^, the displacement rates are well controlled using an Instron® electronic control interface and their WaveMatrix™ dynamic testing software. The force on the bottom piston is monitored using a Lorenz Messtechnik GmbH K11 load cell with a range of 300 kN and an approximate accuracy of 0.05% of the measured force. Surrounding a length of the pistons up to 15 times the length of the sample is a three-zone split furnace from Gero GmbH with a limiting temperature of 1100 °C accurate to within 2 °C. Thermal gradients were minimized by thorough insulation and temperature was recorded at several points and inside the samples with K-type thermocouple arrays. At equilibrium, the hot zone is 0.12 m long. Outside of two cooling rings—and therefore protected from temperature effects—are two piezoelectric AE broadband transducers with 125 kHz central frequency. The AE signals are fed through a 40 dB buffered pre-amplifier, before being recorded at a Richter data acquisition system from Applied Seismology Consultants, which has a 20 MHz sample rate.

### Syn-deformation acoustic emission testing

AE event onsets were triggered and recorded automatically from the continuous acoustic data using an adaptation of an autoregressive-Akaike-Information-Criterion (AR-AIC) picker^50^. The AR-AIC picker follows a defined workflow: (i) detection of the onset of a waveform above the baseline using an STA-LTA detector, (ii) de-noising of the acoustic signal, and (iii) AIC computation where the minimum indicates the arrival time. The STA-LTA window was set to 1 and 20 ms, respectively and the STA/LTA threshold was 2. The amplitude in dB of each single event was also computed (based on a resistance reference standard value of 10 kΩ). The amplitude threshold was set at 68 dB to eliminate noise further. Finally, the acoustic emission data was cut at time of contact and time of failure (or end of the experiment), consistent with the mechanical data, allowing excellent synchronization.

### Materials and samples

Samples were cored from obsidian blocks from Hrafntinnuhryggur, Krafla, Iceland, or pre-prepared from extruded soda-lime-silica glass rods from Schott GmbH, Germany. All samples were cut to 20 mm diameter and an initial 40 mm height, termed *L*_0_. The press was set to use a constant velocity *v* of the upper piston, which was tuned so that the axial strain rate in the sample $\dot \gamma_a = v/L_0$ covered a wide range. Using Eq. 1, and the details in the main text, we predicted *λ*_*r*_, which allowed us to predict what range of $\dot \gamma$ was required to conduct experiments that straddle Wi = 1 by a few orders of magnitude either side for each temperature. We used temperature 550–800 °C for the soda-lime-silica glass, and 750–1000 °C for the obsidian. In the ${\mathrm{Wi}} \ll 1$ regime, we used the Gent^51^ method to confirm our calculated *μ* value, through a direct measurement, validating the method and providing confidence when moving to experiments at Wi > 1. For the soda-lime-silica glass, we found that our measured *μ* agreed well with the temperature-dependent value provided by Schott GmbH, and for the obsidian, we found that *μ* agreed well with the Hess and Dingwell^31^ model for $C_{H_2O} = 0.14$ wt.%, consistent with measurements presented for the same material from the same location^17^. For the obsidian, this confirmed that degassing by diffusion out of the sample during the test was negligible, and we confirmed that bubbles did not nucleate during the test. Mechanical and acoustic data was analysed and visualized using custom-made Python™ algorithms or the ObsPy package^50^.

For data compiled from the literature, we carefully applied the Trouton ratio correction for any value of *σ*, to extract an estimate for *τ* (used in Fig. 5). Otherwise, we used published values of *μ* and the general approximation for *G*_∞_ of 10^10^ Pa quoted in the main text.

## Data Availability

All processed and raw data is available from the corresponding author on request.
